# Mauriac Syndrome Still Exists in Poorly Controlled Type 1 Diabetes: A Report of Two Cases and Literature Review

**DOI:** 10.7759/cureus.14704

**Published:** 2021-04-26

**Authors:** Alya H Alhajjaj, Fatemah K Aljishi

**Affiliations:** 1 Internal Medicine and Endocrinology, Qatif Central Hospital, Qatif, SAU; 2 Internal Medicine, Qatif Central Hospital, Qatif, SAU

**Keywords:** mauriac syndrome, diabetes, hepatomegaly, puberty

## Abstract

Mauriac syndrome is a very rare syndrome that occurs in poorly controlled type 1 diabetes mellitus with diabetic complications. Its cardinal features include delayed growth and puberty, hepatomegaly, and moon faces. These features were attributed mainly to insulin deficiency and sub-optimal diabetic management. Its incidence is decreasing due to the newer insulin formulation and intensive blood glucose control. Early recognition and management of this syndrome may improve the outcome of these patients. Recently, there are increasing reports of this syndrome.

Here, we present the cases of two adolescent males with type 1 diabetes who presented with the classical features of Mauriac syndrome.

## Introduction

Mauriac syndrome (MS) is a very rare syndrome that occurs in poorly controlled type 1 diabetes mellitus (T1DM) with diabetic complications. The cardinal features of MS include delayed growth, delayed puberty, hepatomegaly, and dyslipidemia. Some patients may have cushingoid features and proximal muscle wasting [1,2].

Its exact incidence is not known due to scarcity of reported cases. Most of the reported cases were in children and adolescents, with an equal sex ratio [1]. Since its discovery around 90 years ago, the incidence of MS is decreasing with the use of intensive insulin regimens and the availability of new insulin analogues. In spite of this, there are increasing reported cases of MS [1]. We report two cases with poorly controlled T1DM with the classical features of MS.

We previously presented this article as a meeting poster at the Seventh Annual Clinical Congress of AACE Gulf Chapter Meeting on October 12, 2019.

## Case presentation

Case 1 

A 15-year-old male was referred to our endocrine service in September 2017 with a seven-year history of T1DM. The reason for referral was delayed growth and puberty. The patient was on subcutaneous insulin glargine and premeal insulin aspart. He was noncompliant with his insulin therapy. His baseline glycated hemoglobin (HbA1c) was 10%-13%. His disease course was complicated by frequent admissions for recurrent diabetic ketoacidosis (DKA), diabetic nephropathy, retinopathy, and neuropathy.

At the time of the referral, the patient was admitted with a severe DKA and a staphylococcal bacteremia complicated by abdominal abscesses formation, osteomyelitis, and infective endocarditis. He was managed under the supervision of the infectious diseases service. Upon his evaluation, the patient’s height was 120 cm (<3rd percentile) and weight was 25 kg (<3rd percentile). He had coarse facial features, abdominal distension, hepatomegaly with a liver span of 16 cm, and Tanner stage I for pubic hair and testicular size. His fundus examination showed background diabetic retinopathy.

**Figure 1:**
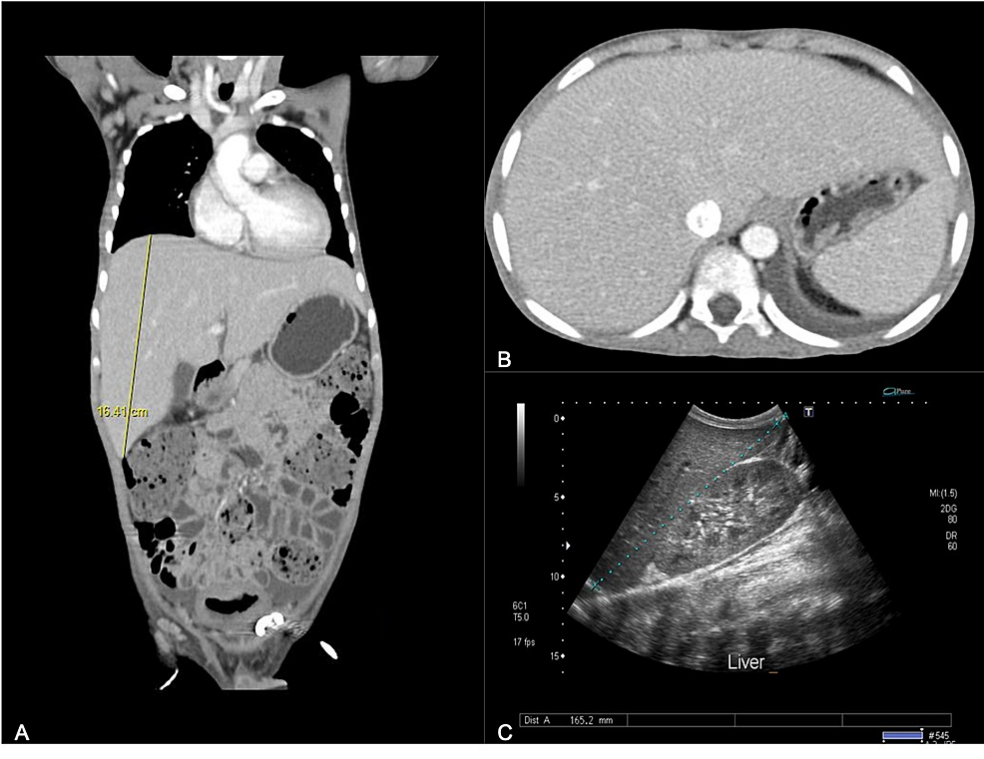
Abdominal imaging of patient in case 1 (A) Coronal CT scan view of the abdomen shows an enlarged liver. (B) Transverse view shows hepatomegaly, peri-portal edema, and moderate ascites with suspected intra-abdominal collections. (C) Longitudinal gray scale ultrasound view shows an enlarged liver measuring 17.3 cm.

His laboratory studies showed microcytic anemia, thrombocytopenia, and low albumin. His iron was 17 ug/dL and the total iron-binding capacity (TIBC) was 84 ug/dL. The patient’s HbA1c was 6.9%. His hepatic panel revealed high alkaline phosphatase (ALP) of 325 U/L (normal range: 40-150 U/L) and gamma-glutamyl transferase (GGT) of 125 U/L (normal range: 5-60 U/L) with normal liver transaminases. Also, he had high triglycerides (TG) of 397 mg/dL. Celiac workup was negative. His hormonal profile revealed insulin-like growth factor 1(IGF-1) of 62.1 ng/dL (normal range: 261-470.8 ng/dL), luteinizing hormone (LH) of 0.32 mIU/mL (normal range: 1.7-8.6 mIU/mL), follicle-stimulating hormone (FSH) of <0.1 mIU/mL (normal range: 1.5-12.1 mIU/mL), and testosterone of 0.22 nmol/L (normal range: 9.9-27.8 nmol/L). Thyroid function test was normal. The 24-hour urine protein collection was 3,876 mg/day. The bone age was 11 years. Because of the severe illness of the patient, growth hormone stimulation tests were not performed. Abdominal ultrasound and CT scan were performed (Figures 1A, 1B).

Based on his clinical presentation and investigations, we diagnosed him with MS. During his admission, the patient’s blood glucose was fluctuating between hyper- and hypoglycemia. His family discharged him against medical advice and he was lost to follow-up. Three months later, the patient was re-admitted again with DKA and septicemia, and, unfortunately, he died.

Case 2 

A 17-year-old male who was referred to our endocrine clinic at the age of 15 years with an eight-year history of a poorly controlled T1DM. Upon referral, he was on insulin glargine and premeal insulin glulisine. He was noncompliant with his insulin therapy. His blood glucose was uncontrolled with hypo-and hyperglycemic levels. His baseline HbA1c ranged from 9% to 11%. There was no history of microvascular complications.

His physical examination revealed a height of 138 cm (<3rd percentile) and weight of 40 kg (<3rd percentile). He had abdominal distension with hepatomegaly, and liver span was 17 cm. His pubic hair and testicular size were Tanner stage 2.

His laboratory tests showed HbA1c of 9.8%, ALP of 200 U/L, aspartate transaminase (AST) of 169.2 U/L (normal range: 0-38 U/L), and alanine transaminase (ALT) of 310.6 U/L (normal range: 10-50 U/L). The cholesterol was 245 mg/dL, low-density lipoprotein (LDL) cholesterol was 144 mg/dL, and TG level was normal. The rest of his metabolic panel was normal. The IGF-1, LH, FSH, and testosterone were low. Thyroid function test was normal. Hepatic serology and autoimmune work-up were negative.

**Figure 2:**
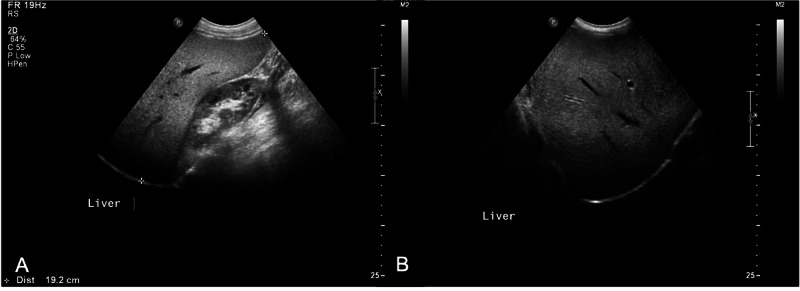
Abdominal ultrasound of patient in case 2 (A) Longitudinal gray scale ultrasound view shows hyperechoic liver (liver steatosis) and normal right kidney. The liver span is enlarged, measuring 19.2 cm in the midclavicular line. (B) Transverse view shows hyperechoic liver.

His bone age was about 14 years (chronological age of 15 years). Abdominal ultrasound revealed enlarged hyperechogenic liver with a size 19.2 cm, suggestive of liver steatosis (Figures 2A, 2B).

Based on his clinical presentation and investigations, we diagnosed him with MS. He was put on an intensive insulin regimen. His blood glucose improved and liver enzymes normalized. Clinically, his liver size shrunk significantly. During his follow-up, he got his puberty, and his final height was 146 cm and weight was 46 kg.

## Discussion

In 1930, Pierre Mauriac described MS in a 10-year-old girl with poorly controlled T1DM [3]. Since that time, several cases of MS have been published. It is more common in patients with poorly controlled diabetes with evidence of diabetic complications such as retinopathy and nephropathy, similar to our first case [4].

These features of MS are most likely related to insulin deficiency [5] and can be improved with the intensification of insulin therapy [2]. As in our second case, with improvement in blood glucose control, the patient's liver enzymes normalized and his liver size decreased.

MS can be categorized into two different forms according to the presence or absence of obesity [2]. The first MS form includes patients with obesity and cushingoid features. Elevated corticosteroids resulting from variable plasma glucose levels are the most likely responsible cause for these features. On the other hand, the second MS form includes patients who are not obese with persistent hyperglycemia [2]. Both of our cases had the second form of MS.

The etiology of delayed growth in MS is multi-factorial. These factors include inadequate tissue glucose, decreased growth hormone levels and IGF-1, impaired or resistant hormone receptor action, and hypercortisolism [1,6]. The hepatomegaly and impaired liver function are thought to be due to glycogen deposition in hepatocytes (hepatic glycogenosis [HG]) [6].

The cause of HG is due to alternating periods of hyperglycemia and hyperinsulinemia. Hyperinsulinemia resulting from insulin administration stimulates the synthesis of glycogen from excessive hepatic glucose [7]. Histological features of HG are similar to glycogen storage disease. These features include glycogen accumulation and glycogenated nuclei [7]. Some investigators suggested that a genetic mutation in an enzyme of glycogen metabolism combined with persistent hyperglycemia may be the cause of hepatomegaly in MS [8].

## Conclusions

In spite of advancement in diabetic management, MS - a rare complication in poorly controlled T1DM - still exists. A high index of suspicion is needed in T1DM with delayed growth and puberty since good metabolic control could reverse this rare condition.
